# Two *Lactobacillus* Species Inhibit the Growth and α-Toxin Production of *Clostridium perfringens* and Induced Proinflammatory Factors in Chicken Intestinal Epithelial Cells *in Vitro*

**DOI:** 10.3389/fmicb.2017.02081

**Published:** 2017-10-25

**Authors:** Shuangshuang Guo, Dan Liu, Beibei Zhang, Zhui Li, Yehan Li, Binying Ding, Yuming Guo

**Affiliations:** ^1^State Key Laboratory of Animal Nutrition, College of Animal Science and Technology, China Agricultural University, Beijing, China; ^2^Hubei Key Laboratory of Animal Nutrition and Feed Science, Hubei Collaborative Innovation Center for Animal Nutrition and Feed Safety, Wuhan Polytechnic University, Wuhan, China

**Keywords:** *Lactobacillus*, *Clostridium perfringens*, α-toxin, chicken, intestinal epithelial cell, inflammation

## Abstract

*Clostridium perfringens* is the causative pathogen of avian necrotic enteritis. *Lactobacillus* spp. are well-characterized probiotics with anti-microbial and immune-modulatory activities. In the present study, we investigated the effects of *L. acidophilus* and *L. fermentum* on the growth, α-toxin production and inflammatory responses of *C. perfringens*. In *in vitro* culture experiments, both lactobacilli inhibited the growth of *C. perfringens* (*P* < 0.01), accompanied with a decrease in pH (*P* < 0.01). Supernatants from lactobacilli cultures also suppressed the growth of *C. perfringens* during 24 h of incubation (*P* < 0.01), but this inhibitory effect disappeared after 48 h. Both lactobacilli decreased the α-toxin production of *C. perfringens* (*P* < 0.01) without influencing its biomass, and even degraded the established α-toxin (*P* < 0.01). Lower environmental pH reduced the α-toxin production as well (*P* < 0.01). Preincubation with *L. acidophilus* decreased the attachment of *C. perfringens* to cells (*P* < 0.01) with the cell cytotoxicity being unaffected. Both lactobacilli pretreatment reduced the up-regulation of proinflammatory factors, peptidoglycan (PGN) receptors and nuclear factor kappa B (NF-κB) p65 in *C. perfringens*-challenged chicken intestinal epithelial cells (*P* < 0.05). In conclusion, *L. acidophilus* and *L. fermentum* inhibited the pathological effects of *C. perfringens in vitro* conditions.

## Introduction

*Clostridium perfringens* is a robust Gram-positive anaerobic spore-forming and rod-shaped bacterium, and able to produce up to 18 toxins and extracellular enzymes, causing diseases, such as necrotic enteritis (NE) in poultry, equine colitis, food poisoning, and gas gangrene (Songer and Meer, 1996; Revitt-Mills et al., 2015). *C. perfringens* strains are classified into five toxinotypes (A, B, C, D, and E) based on the production of four major toxins (α, β, ε, and ι) (Petit et al., 1999). Type A strains are the most widespread in the intestines of warm-blooded animals and in the environment, mainly responsible for avian NE (Songer, 1996). In 2015, the costs of NE came close to USD six billion (Wade and Keyburn, 2015).

The α-toxin is produced by all five types of *C. perfringens* and belongs to a family of bacterial zinc-metallo phospholipase C enzymes. Although α-toxin is not the essential virulence factor to cause NE (Keyburn et al., 2006), it might still be important in the pathogenesis. It was reported that α-toxin was detected in the intestines of broiler chickens inoculated with a α-toxin mutant strain of *C. perfringens* and there was a direct relationship between intestinal lesion severity and amount of α-toxin determined in the gut contents and mucosa (*R* = 0.89–0.99) (Coursodon et al., 2010). Furthermore, α-toxin is a key mediator of human gas gangrene and bovine necro-haemorrhagic enteritis (Bryant et al., 2000; Goossens et al., 2017). It could result in myonecrosis, hemorrhage and neutrophil infiltration in human and animals.

Microscopic examination of early stages of NE shows strong intestinal inflammation to *C. perfringens* infection (Timbermont et al., 2011). The lamina propria of intestine is hyperaemic and infiltrated with numerous inflammatory cells, mainly heterophilic granulocytes. Moreover, the gene expression of cytokines, such as interferon (IFN)-γ, interleukin (IL)-1β, and IL-10 was significantly up-regulated in the intestine of NE chickens (Collier et al., 2008; Park et al., 2008). Using *in vitro* primary culture of chicken intestinal epithelial cells, we previously demonstrated that both *C. perfringens* and α-toxin challenge induced intense cytokine expression by activating the nuclear factor kappa B (NF-κB) signaling pathway (Guo et al., 2015). Furthermore, the cell receptors, Toll-like receptor (TLR) 2 and nucleotide-binding oligomerization domain (NOD) 1, might play a role in the recognition of peptidoglycan (PGN) component of *C. perfringens* (Lu et al., 2009; Guo et al., 2015).

*Lactobacillus* spp. are members of the normal intestinal flora and used as probiotics to prevent the growth and colonization of *C. perfringens* in NE model of poultry (La Ragione et al., 2004; Rahimi et al., 2011). It was reported that orally administration of *Lactobacillus fermentum* strain 1.2029 reduced the inflammatory damage of NE in chickens by up-regulating IL-10 mRNA levels and down-regulating the mRNA expression of IFN- γ and TLR2 (Cao et al., 2012). In a co-infection model with *Eimeria* spp. and *C. perfringens*, broiler chickens fed *L. johnsonii* had lower NE lesion scores and improved feed efficiency (Geier et al., 2010). *In vitro* co-culture system, *L. fermentum* strain 104R silenced *C. perfringens* β2 toxin production by decreasing *cpb2* mRNA, which was most likely a result of the decline in environmental pH (Allaart et al., 2011). However, the effect of *Lactobacillus* spp. on the α-toxin production and inflammation of *C. perfringens* as well as the underlying mechanisms are largely unknown. The purpose of this study was to investigate the effects of *Lactobacillus acidophilus* and *Lactobacillus fermentum* on the growth and α-toxin production of *C. perfingens* as well as the inflammatory responses induced by this pathogen in the intestinal epithelial cells of chicken embryos.

## Materials and methods

### Materials

All experimental procedures used were approved by the China Agricultural University Animal Care and Use Committee. Bacterium culture media and agars were obtained from Beijing Land Bridge Technology Co. Ltd. (Beijing, China). Cell culture media, buffers and fetal bovine serum (FBS) were purchased from Gibco (Carlsbad, CA, USA). Epidermal growth factor (EGF) was purchased from BD Biosciences (San Jose, CA, USA). Bacterium and cell culture plastics were obtained from Corning Life Science (Tewksbury, MA, USA). All other chemicals, unless specified, were obtained from Sigma-Aldrich (St. Louis, MO, USA).

### Bacterial strains and culture conditions

*Lactobacillus acidophilus* (CGMCC No. 1.1878) and *L. fermentum* (CGMCC No. 1.2029) were obtained from China General Microbiological Culture Collection Center (CGMCC, Beijing, China), and cultured in Mann-Rogosa-Sharpe (MRS) broth. The chicken *C. perfringens* type A field strain (CVCC No. 2030) was obtained from China Veterinary Culture Collection Center (CVCC, Beijing, China) and cultured in cooked meat medium. Both lactobacilli and *C. perfringens* were incubated at 37°C under anaerobic conditions. All bacterial experiments were performed with MRS broth, since this provided the best overall growth environment for all species (Allaart et al., 2011). For cell experiments, overnight-incubated bacterial cultures were centrifuged at 4,000 × *g* for 10 min, washed twice by sterile phosphate buffered saline (PBS, pH 7.4) and resuspended in cell culture medium. Bacterial concentration was estimated by measuring the absorbance at 600 nm and by relating the optical density to colony forming units (CFU, data not shown).

### Lactobacilli and *C. perfringens* co-culture experiments

The procedures for bacterial co-culture experiments used in the current study were previously described by Jiang et al. (2014) with some modifications. Strains of lactobacilli and *C. perfringens* were cultured individually under anaerobic conditions in MRS broth and cooked meat medium, respectively, overnight to reach a stationary phase. Both cultures were centrifuged at 4,000 × *g* for 10 min. The pellet was then washed once with PBS and resuspended in fresh MRS broth with a concentration of 1 × 10^7^ CFU/mL. Thirty microliters of *L. acidophilus* and *L. fermentum* suspensions were individually inoculated into 3 mL of fresh MRS broth additionally containing 30 μL of *C. perfringens* suspension. Therefore, the ratio of *Lactobacilli* to *C. perfringens* was 1:1 in a 3.06 mL co-culture system. Another 30 μL suspensions of *L. acidophilus, L. fermentum*, and *C. perfringens* were individually added to the 3.03 mL of fresh MRS broth. This experiment was performed in triplicate. Cultures were anaerobically incubated at 37°C for 20 h. Following incubation, CFU of lactobacilli and *C. perfringens* were determined on MRS agar plates and tryptose-sulfite-cycloserine (TSC) agar plates, respectively. Meanwhile, the pH of cultures was measured.

### Agar well diffusion assay and broth culture inhibition assay

The preparation of cell-free supernatant from lactobacilli cultures and agar well diffusion assay as well as broth culture inhibition assay was performed as previously described (Schoster et al., 2013), with some modifications. Briefly, lactobacilli suspensions were adjusted to an optical density of 0.220 at 600 nm and 1 mL of this suspension was inoculated into 9 mL of MRS broth. After 24 and 48 h of incubation, the lactobacilli cultures were centrifuged at 4,000 × *g* for 10 min and the supernatant was collected, filter-sterilized using a 0.22 μm membrane syringe filter and divided into two aliquots, one of which was neutralized to pH 6.20 using NaOH. Both aliquots were checked for absence of viable bacteria by plating on MRS agar and then frozen at −80°C for further use.

Overnight culture of *C. perfringens* was diluted 10-fold up to 10^−2^ to achieve confluent growth. When the autoclaved TSC medium cooled down to about 55°C, 100 μL of bacterial dilution were mixed well with 20 mL TSC medium in a 10-cm plastic plate. Five 9-mm wells were made in each agar plate and 100 μL of lactobacilli supernatants were added to the four outer wells whereas sterile MRS broth was put into the central well (control well). When the effect of lactobacilli supernatants without neutralization was evaluated, MRS broth adjusted to pH 4.20 (using HCl) was added to the control well. When using neutralized supernatants, MRS broth at pH 6.20 was used instead. The presence of an inhibition zone >1 mm was assessed visually following 16 h of anaerobic incubation at 37°C. Each assay was performed in duplicate.

Fifty microliters of *C. perfringens* suspension were added to 5 mL of fresh MRS broth and 1 mL of lactobacilli supernatants harvested after 48 h of incubation. MRS broth at pH 6.20 and pH 4.20 was used as controls for the supernatant with and without pH neutralization, respectively. Growth was measured spectrophotometrically at 0, 24 and 48 h after anaerobic incubation at 37°C. The differences of OD_600_ measurements between 0 h of incubation and 24 or 48 h of incubation were calculated. Each assay was performed in triplicate.

### The effect of lactobacilli on *C. perfringens* α-toxin production

Fifteen milliliters of *C. perfringens* cultures in a stationary phase were centrifuged at 4,000 × *g* for 10 min and washed once with PBS and resuspended in the same volume of fresh MRS broth. This suspension was divided into three equal parts. Two of them were supplemented with the bacterial pellets of *L. acidophilus* and *L. fermentum*, respectively. The suspension without lactobacilli pellets was used as the control. After 4 h of anaerobic incubation at 37°C, the numbers of CFU of *C. perfringens* were determined on TSC agar plates and the pH was measured. The cultures were centrifuged at 4,000 × *g* for 10 min, and α-toxin level in the supernatants was analyzed.

As lactobacilli could greatly decrease the pH of cultures, we examined α-toxin production at normal and lower pHs. Pellets from stationary culture of *C. perfringens* were resuspended in MRS broth set at pH 5.20 or pH 6.20 using HCl and incubated anaerobically at 37°C for 2 or 4 h. After each time period, the numbers of CFU of *C. perfringens* were determined and the pH of each culture was measured. The supernatants of cultures were collected for α-toxin assay. All experiments were performed in triplicate.

### *C. perfringens* α-toxin degradation by lactobacilli

Pellets from stationary culture of *C. perfringens* were resuspended in the same volume of fresh MRS broth and incubated anaerobically at 37°C for 4 h. The supernatant of this culture were collected by centrifugation (4,000 × *g*, 10 min) and divided into three equal parts. Pellets of *L. acidophilus* and *L. fermentum* cultures were individually resuspended in two parts of this supernatant and incubated anaerobically at 37°C for 2 or 4 h. The culture without lactobacilli pellets was used as the control. At each time point, the pH of each culture was measured and the supernatants of cultures were sampled for α-toxin assay. This experiment was performed in triplicate.

### Elisa assay of *C. perfringens* α-toxin

The content of *C. perfringens* α-toxin in the supernatants of cultures was determined using antigenic ELISA kit (Bio-X Diagnostics, Rochefort, Belgium), according to the manufacturers' instructions. Each sample and the control antigen (supplied by the kit) were tested in both positive and negative wells. The data were expressed as a percentage of delta optical density of each sample (between positive and negative wells) to that of the control antigen.

### Primary culture of chicken intestinal epithelial cells

The methods of isolating primary intestinal epithelial cells from chicken embryos were previously described in detail by Guo et al. (2015). Isolated cells were grown in a 1:1 mixture of Dulbecco's modified Eagles medium and Ham's F12 medium (DMEM/F12) supplemented with 2.5% FBS, 100 U/mL penicillin, 100 μg/mL of streptomycin, 20 ng/mL of EGF, 100 μg/mL of heparin sodium salt, 5 μg/mL of insulin. Cells were maintained in a humidified environment with an atmosphere of 5% CO_2_ at 37°C.

### Cell adhesion and cytotoxicity assay

The assay of bacterial adhesion to cells was performed as previously described (Martin and Smyth, 2010) with some modification. The isolated cells were seeded in 12-well cell culture plates at a density of 1.0 × 10^6^ cells/mL with a total volume of 1 mL. After 48 h of incubation, cells were gently washed with pre-warmed PBS. One milliliter of DMEM/F12 medium containing *L. acidophilus* or *L. fermentum* with multiplicity of infection (MOI) at 10 was added to wells. Cells without lactobacilli were used as the control. Following 3 h of anaerobic incubation, a few microliters of resuspended *C. perfringens* was inoculated into each well (MOI = 1) and incubated at 37°C under anaerobic condition for 1 h. All experiments were performed in triplicate. After incubation, the cells were washed twice with PBS and 200 μL of 0.05% trypsin was added to each well and incubated for 15 min at 37°C. An additional 800 μL of PBS was added to each well and mixed gently by pipetting. The number of *C. perfringens* released per well was determined by plating serial 10-fold dilutions of the cell suspension onto TSC plates. The percentage of adhering bacteria was calculated by dividing the number of bacteria recovered from wells with the total number of bacteria added to the wells. Meanwhile, the remaining cell suspension were centrifuged at 4,000 × *g* for 10 min and the supernatant was collected for cytotoxicity quantification. Lactate dehydrogenase (LDH), an enzyme present in the cytoplasm of cells, is quickly resealed upon damage of the plasma membrane. The LDH production was determined using commercially available kit (Nanjing Jiancheng Biological Product, Nanjing, Jiangsu Province, China) following the manufacturer's protocol.

### Inflammatory responses

Cells were seeded in 6-well cell culture plates at a concentration of 1.0 × 10^6^ cells/mL (2 mL per well). At 90% confluence, cells were pretreated with either *L. acidophilus* or *L. fermentum* at MOI of 1 for 2 h, and then stimulated with and without *C. perfringens* (MOI = 0.1) for 6 h. The DMEM/F12 medium and *C. perfringens* infection alone served as the negative and positive controls, respectively. Experiments were run in triplicate. After stimulation, the supernatant of cell culture medium was collected for the assay of LDH activity and cells were lysed for RNA extraction.

### RNA isolation and quantitative real-time PCR

The methods of RNA extraction and quantitative real-time PCR were described in our previous publication (Guo et al., 2015). Total RNA of the cells was extracted using TRIzol reagent (Invitrogen Life Technologies, Carlsbad, CA, USA) according to the manufacturer's instructions. The concentration and purity of total RNA was determined by measuring its optical density at 260 and 280 nm, and the RNA integrity was assessed via agarose gel electrophoresis. One microgram of total RNA was reverse transcribed with PrimeScript^TM^ RT Master Mix (Perfect Real Time) (TakaRa, Dalian, Liaoning Province, China). All cDNA preparations were stored frozen at −20°C until further use. The qRT-PCR analysis was performed with the 7,500 fluorescence detection system (Applied Biosystems, Foster City, CA) using the SYBR® *Premix Ex Taq*^TM^ (TaKaRa). The primer pairs for the amplification of chicken IL-6, IL-8, lipopolysaccharide-induced tumor necrosis factor-alpha factor (LITAF), inducible nitric oxide synthase (iNOS), IL-1β, NOD1, TLR2.2, TLR4, NF-κB p65, and β-actin cDNA fragments, used as an endogenous reference gene, were used as listed in Table 1. To confirm amplification specificity, the PCR products from each primer pair were subjected to a melting analysis and subsequent agarose gel electrophoresis. Samples were run in triplicate in 96-well plates. Relative gene expression data were analyzed using the 2^−ΔΔCt^ method (Heid et al., 1996).

**Table 1:** Primers used for quantitative real-time PCR.

**Gene name**	**Accession number**	**Forward sequence (5′-3′)**	**Reverse sequence (5′-3′)**
β-actin	L08165	GAGAAATTGTGCGTGACATCA	CCTGAACCTCTCATTGCCA
IL-6	AJ309540	CAAGGTGACGGAGGAGGAC	TGGCGAGGAGGGATTTCT
IL-8	AJ009800	ATGAACGGCAAGCTTGGAGCTG	TCCAAGCACACCTCTCTTCCATCC
iNOS	U46504	CAGCTGATTGGGTGTGGAT	TTTCTTTGGCCTACGGGTC
LITAF	NM_204267	GAGCGTTGACTTGGCTGTC	AAGCAACAACCAGCTATGCAC
IL-1β	NM_204524	ACTGGGCATCAAGGGCTA	GGTAGAAGATGAAGCGGGTC
TLR2.2	NM_001161650	GGGGCTCACAGGCAAAATC	AGCAGGGTTCTCAGGTTCACA
NOD1	JX465487	AGCACTGTCCATCCTCTGTCC	TGAGGGTTGGTAAAGGTCTGCT
NF-κB p65	NM_205129	GTGTGAAGAAACGGGAACTG	GGCACGGTTGTCATAGATGG

*IL, interleukin; iNOS, inducible nitric oxide synthase; LITAF, lipopolysaccharide induced TNF factor; TLR, toll-like receptor; NOD, nucleotide-binding oligomerization domain; NF-κB, nuclear factor kappa B*.

### Statistical analysis

All data were analyzed with SPSS Version 17.0 (SPSS Inc., Chicago, IL). For the data of inflammatory responses, the comparison between negative and positive controls was conducted using an independent-samples *t*-test. The other data were subjected to one-way analysis of variance (ANOVA) and Tukey test was used for multiple comparisons between treatments. Significance was accepted at *P* < 0.05 and results were reported as means and standard error (SE).

## Results

### Lactobacilli and their supernatants inhibit the growth of *C. perfringens*

**Figure 1:**
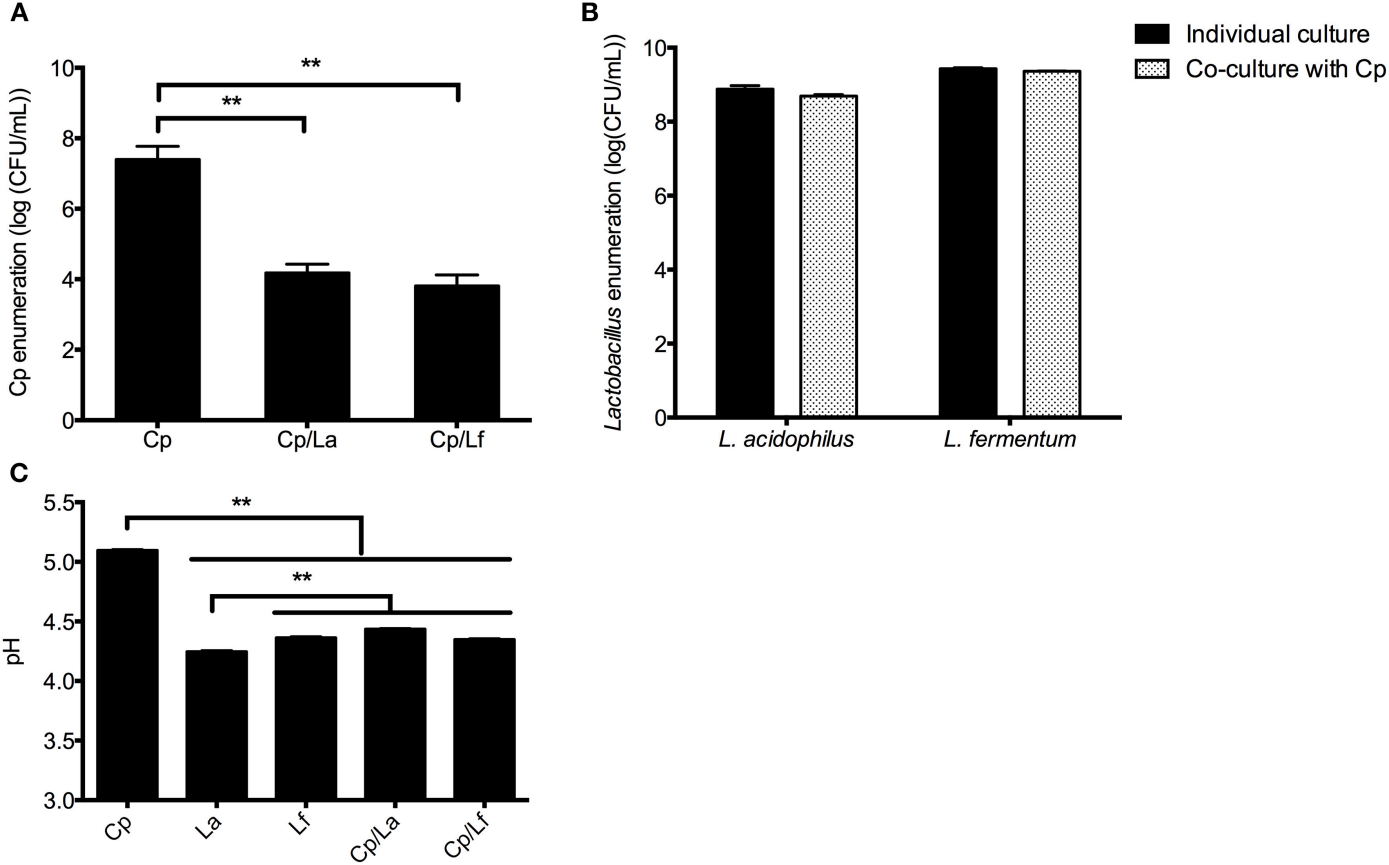
Bacterial enumeration and pH measurement in the co-culture experiment. Lactobacilli and *C. perfringens* were individually or together inoculated into the MRS broth with equal CFU and cultured at 37°C for 20 h. The enumeration of *C. perfringens* **(A)** and lactobacilli **(B)** as well as pH values of cultures **(C)** were determined at the end of incubation. Data are presented as mean ± standard error (*n* = 3). ^**^P < 0.01. La, *L. acidophilus*; Lf, *L. fermentum*; Cp, *C. perfringens*.

Compared with culture alone, co-culture of *C. perfringens* with either *L. acidophilus* or *L. fermentum* decreased the number of *C. perfringens* after 20 h of incubation (*P* < 0.01; Figure 1A), but did not significantly affect the enumeration of both lactobacilli (Figure 1B). Unsurprisingly, lactobacilli growth, alone or together with *C. perfringens*, reduced the pHs of cultures in comparison with individual *C. perfringens* culture (*P* < 0.01; Figure 1C).

**Figure 2:**
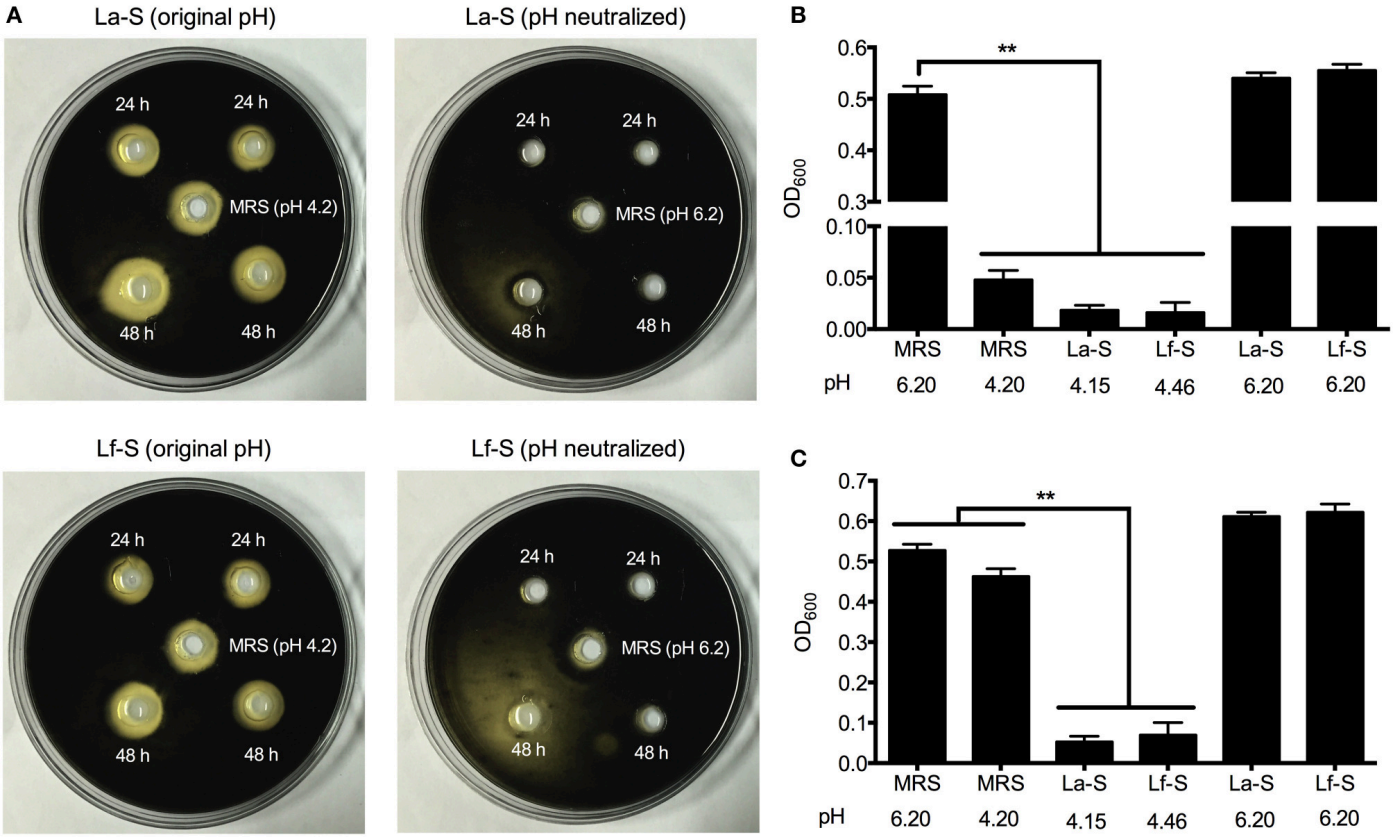
Inhibition of *C. perfringens* by cell-free supernatants with and without pH neutralization, obtained from *L. acidophilus* and *L. fermentum*. Agar well diffusion assay was performed and the inhibition zone was assessed visually **(A)**. Lactobacilli supernatants were collected after 24 or 48 h of incubation. Central wells were filled with sterile MRS broth adjusted to pH 4.2 or standard MRS broth. The colonies of *C. perfringens* were black in the TSC agar. The growth of *C. perfringens* in fresh MRS broth supplemented with lactobacilli supernatants was measured by OD_600_ after 24 **(B)** and 48 **(C)** hours of incubation. Data are presented as mean ± standard error (*n* = 3). ^**^P < 0.01. La-S, supernatants from *L. acidophilus* cultures; Lf-S, supernatants from *L. fermentum* cultures; MRS, Mann-Rogosa-Sharpe medium.

The data of agar well diffusion assay showed that lactobacilli supernatants from 24 to 48 h-cultures exhibited inhibition zones to the growth of *C. perfringens* (Figure 2A). However, when the pHs of supernatants were neutralized, the inhibition zones disappeared. Furthermore, the MRS broth with pH adjusted to 4.20 also exerted inhibitory effect on *C. perfringens* growth. Consistently, addition of both lactobacilli supernatants and MRS broth (pH 4.20) to *C. perfringens* cultures significantly decreased the OD_600_ values (*P* < 0.01), which reflected the number of *C. perfringens*, following 24 h of incubation (Figure 2B). As the incubation time reached 48 h, the lactobacilli supernatants still suppressed the OD_600_ values of cultures compared with the control (MRS broth at pH 6.20) (*P* < 0.01), but no significant differences were observed between the control and MRS broth at pH 4.20 (Figure 2C).

### Lactobacilli repress the α-toxin production of *C. perfringens*

**Figure 3:**
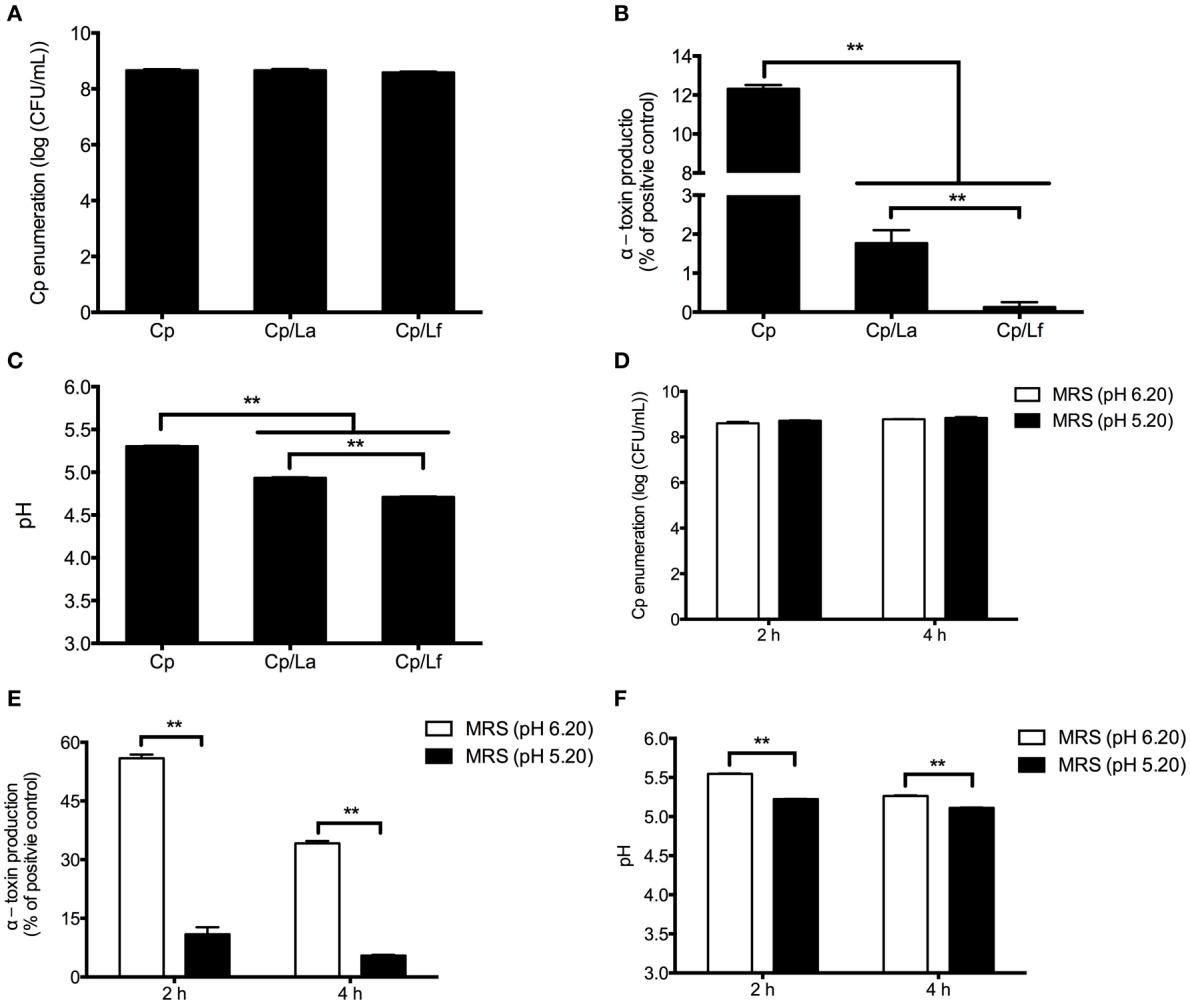
The effect of lactobacilli and pH on the number and α-toxin production of *C. perfringens* as well as pH of cultures. *C. perfringens* enumeration **(A)**, α-toxin production **(B)** and pH values **(C)** were determined after 4-h co-culture of *C. perfringens* and lactobacilli. *C. perfringens* was grown in the MRS broth with normal (6.20) and lower (5.20) pHs for 2 and 4 h. The CFU of *C. perfringens* **(D)**, α-toxin secretion **(E)** and pH **(F)** were measured at each time point. Data are presented as mean ± standard error (*n* = 3). ^**^P < 0.01. La, *L. acidophilus*; Lf, *L. fermentum*; Cp, *C. perfringens*; MRS, Mann-Rogosa-Sharpe medium.

Following 4 h of incubation, *L. acidophilus* and *L. fermentum* did not significantly influence the number of *C. perfringens* in the co-culture systems (Figure 3A), but decreased the α-toxin secretion compared with *C. perfringens* cultured alone (*P* < 0.01; Figure 3B). What's more, *L. fermentum* showed a greater inhibitory effect on α-toxin production than *L. acidophilus*. The pHs of cultures exhibited similar changes as α-toxin production. Both lactobacilli reduced the pHs of co-cultures and *L. fermentum* induced a further decrease of pH compared with *L. acidophilus* (*P* < 0.01; Figure 3C).

As shown in Figure 3D, the number of *C. perfringens* was not significantly affected by MRS broth at a lower pH (5.20) compared with normal pH (6.20) after 2 and 4 h of culture. However, the α-toxin secretion was impaired by the MRS broth with pH 5.20 following incubation (*P* < 0.01; Figure 3E). As expected, the MRS broth at pH 5.20 maintained a lower level of pH along the treatment (*P* < 0.01; Figure 3F).

### Lactobacilli degrade established *C. perfringens* α-toxin

**Figure 4:**
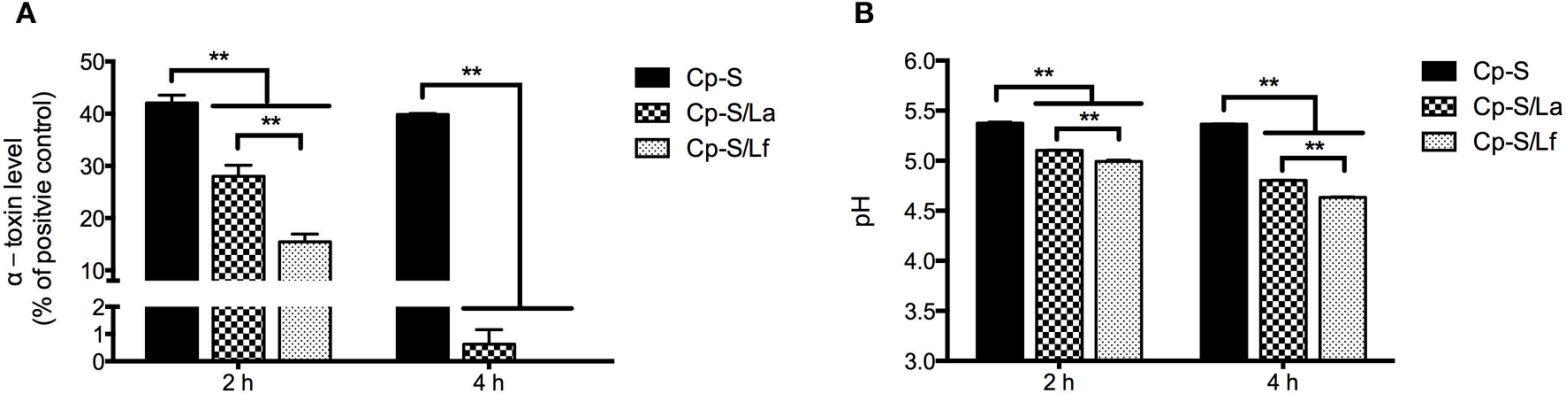
The degradation of *C. perfringens* α-toxin by lactobacilli and associated changes of culture pH. Either *L. acidophilus* or *L. fermentum* were added into the supernatants from *C. perfringens* cultures and incubated for 2 or 4 h. The α-toxin levels were detected by ELISA assay **(A)** and pH values were measured **(B)**. Data are presented as mean ± standard error (*n* = 3). ^**^P < 0.01. La, *L. acidophilus*; Lf, *L. fermentum*; Cp-S, supernatants from *C. perfringens* cultures.

As shown in Figure 4A, the addition of *L. acidophilus* and *L. fermentum* to the supernatants from *C. perfringens* cultures significantly reduced the levels of α-toxin after 2 and 4 h of incubation (*P* < 0.01). And *L. fermentum* resulted in a greater reduction of α-toxin compared to *L. acidophilus* at 2 h of treatment. During incubation, both lactobacilli significantly decreased the pH of *C. perfringens* supernatants in contrast to the control (*P* < 0.01) and lower pH was detected in the *L. fermentum*-treated group in comparison with *L. acidophilus* treatment (*P* < 0.01; Figure 4B).

### The effect of lactobacilli on the adhesion of *C. perfringens* to cells

**Figure 5:**
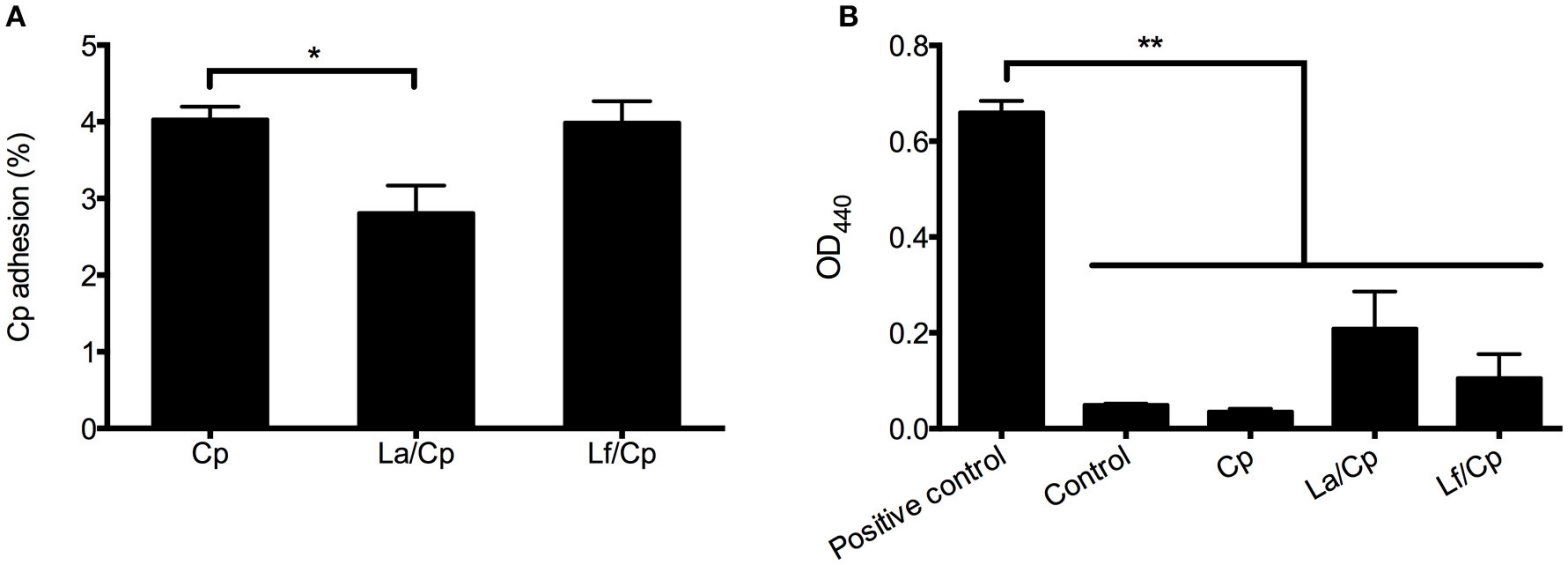
The effect of lactobacilli on the adhesion of *C. perfringens* to cells and the cytotoxicity induced by *C. perfringens*. Cells were pretreated with *L. acidophilus* or *L. fermentum* with multiplicity of infection (MOI) at 10 for 3 h and then infected with *C. perfringens* (MOI = 1) for 1 h. The percentage of *C. perfringens* adhered to cells were calculated **(A)** and LDH levels in the cell culture medium were detected **(B)**. Data are presented as mean ± standard error (*n* = 3). ^*^P < 0.05; ^**^P < 0.01. La, *L. acidophilus*; Lf, *L. fermentum*; Cp, *C. perfringens*.

Compared with *C. perfringens* infection alone, *L. acidophilus* pretreatment significantly decreased the percentage of *C. perfringens* adhering to chicken intestinal epithelial cells (*P* < 0.05), but *L. fermentum* did not show inhibitory effect on the *C. perfringens* adhesion (Figure 5A). The levels of LDH released from cells were not significantly changed after 1 h of incubation with *C. perfringens* alone or together with 3 h of lactobacilli pretreatments (Figure 5B). The LDH levels in the bacterial treatments were also not significantly different from that in the control (cell medium alone) (*P* > 0.05), but lower than that in the positive control, in which all cells were lysed by 0.5% of Triton X-100 (*P* < 0.01).

### The anti-inflammatory effect of lactobacilli in the *C. perfringens*-infected cells

**Figure 6:**
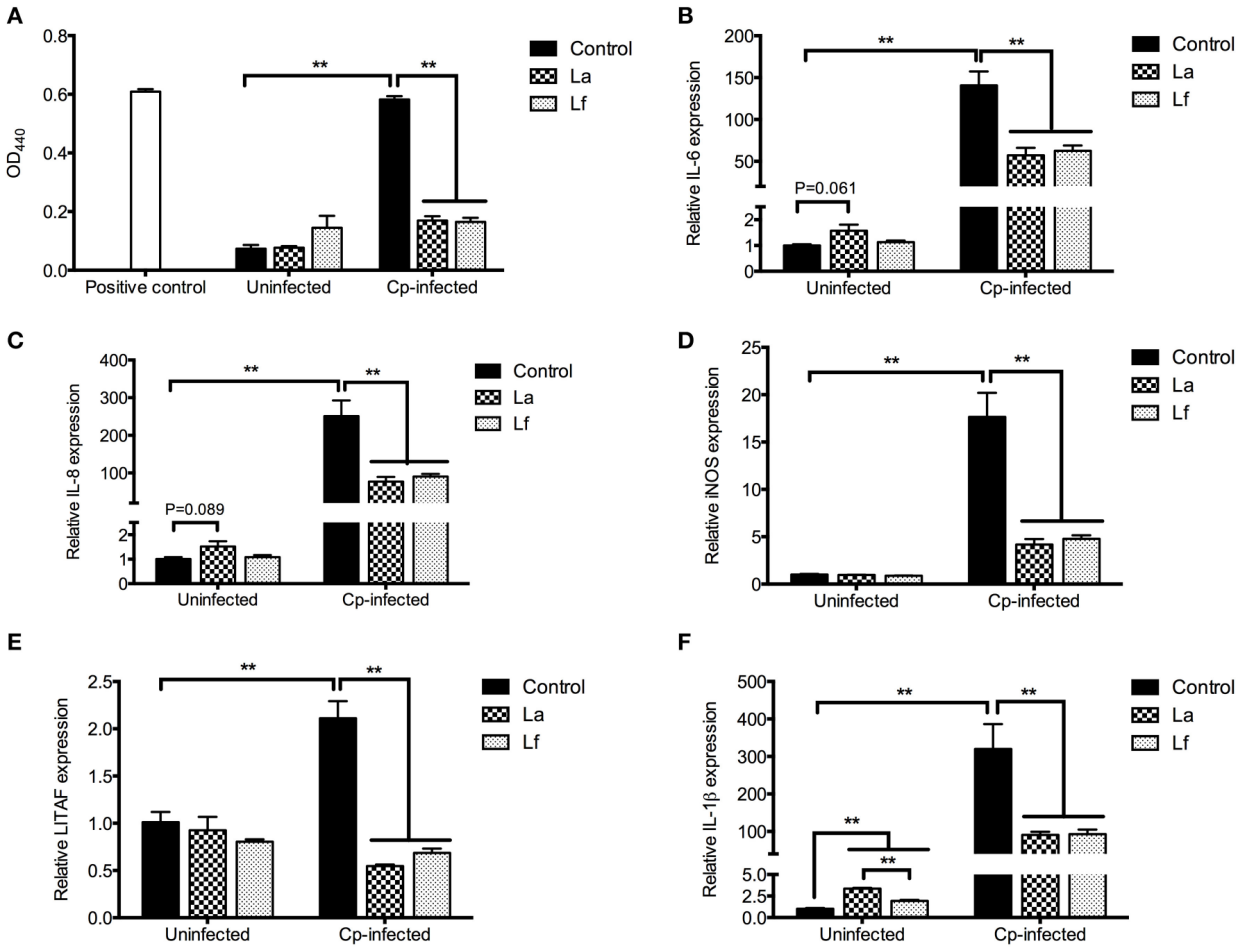
The cytotoxicity and cytokine mRNA expression in the *C. perfringens*-infected cells pretreated with lactobacilli. Cells were pre-incubated with *L. acidophilus* or *L. fermentum* (MOI = 1) for 2 h and then stimulated with *C. perfringens* (MOI = 0.1) for 6 h. Following infection, LDH released from cells to culture medium were detected **(A)**. The mRNA expression of IL-6 **(B)**, IL-8 **(C)**, iNOS **(D)**, LITAF **(E)** and IL-1β **(F)** were determined by qRT-PCR analysis. Data are presented as mean ± standard error (*n* = 3). ^**^P < 0.01. La, *L. acidophilus*; Lf, *L. fermentum*; Cp, *C. perfringens*; IL, interleukin; iNOS, inducible nitric oxide synthase; LITAF, lipopolysaccharide induced TNF factor.

The data of LDH level and cytokine mRNA expression were shown in Figure 6. Compared with the uninfected control, *C. perfringens* infection at MOI of 0.1 for 6 h significantly increased the LDH level released from cells (*P* < 0.01), which indicated the induction of cytotoxicity by infection. However, pre-incubation of *L. acidophilus* and *L. fermentum* at MOI of 1 for 2 h reduced LDH levels in the infected cells (*P* < 0.01). Consistently, the relative mRNA expression of IL-6, IL-8, iNOS, LITAF and IL-1β was up-regulated by the *C. perfringens* challenge (*P* < 0.01), but down-regulated in challenged cells by pretreatment with *Lactobacilli* (*P* < 0.01). In the uninfected cells, both lactobacilli significantly increased the IL-1β mRNA level (*P* < 0.01), and *L. acidophilus* pre-incubation tended to elevate the mRNA expression of IL-6 (*P* = 0.061) and IL-8 (*P* = 0.089).

**Figure 7:**
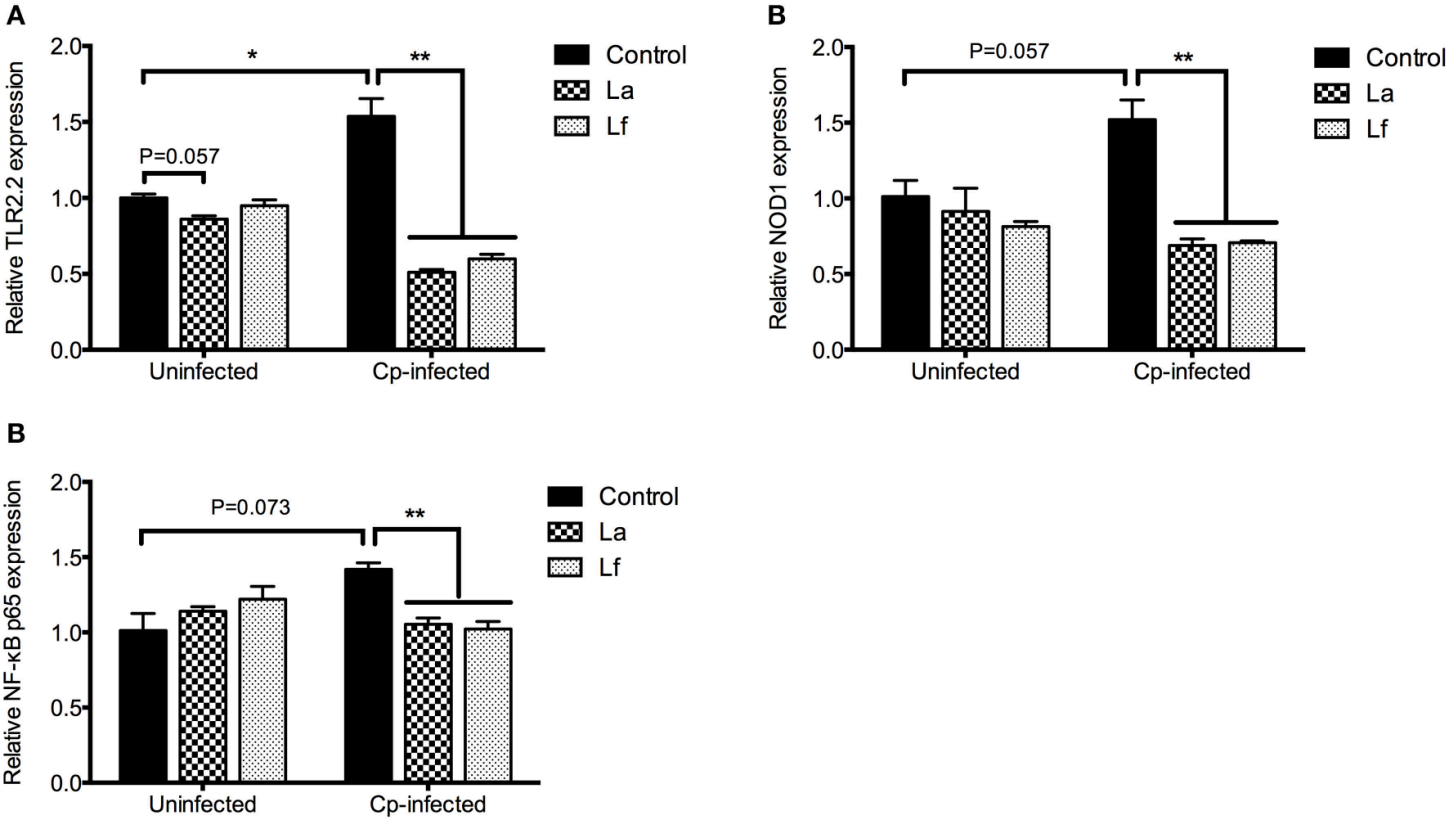
Quantification of TLR2.2, NOD1 and NF-κB p65 transcripts in the cells. Cells were pre-incubated with *L. acidophilus* or *L. fermentum* (MOI = 1) for 2 h and then stimulated with *C. perfringens* (MOI = 0.1) for 6 h. Following infection, the mRNA expression of TLR2.2 **(A)**, NOD1 **(B)** and NF-κB p65 **(C)** were analyzed by qRT-PCR. Data are presented as mean ± standard error (*n* = 3). ^*^P < 0.05, ^**^P < 0.01. La, *L. acidophilus*; Lf, *L. fermentum*; Cp, *C. perfringens*; TLR, toll-like receptor; NOD, nucleotide-binding oligomerization domain; NF-κB, nuclear factor kappa B.

To investigate the possible mechanism mediating the modulation of cytokine expression by lactobacilli pretreatment, the mRNA expression of receptors (TLR2.2 and NOD1) and NF-κB p65 was determined and the data were presented in Figure 7. The *C. perfringens* infection significantly up-regulated the TLR2.2 mRNA expression (*P* < 0.05) and tended to increase the mRNA levels of NOD1 (*P* = 0.057) and NF-κB p65 (*P* = 0.073). However, the relative expression of TLR2.2, NOD1, and NF-κB p65 in the infected cells was reduced by the preincubation of lactobacilli (*P* < 0.01). There was a tendency that *L. acidophilus* decreased TLR2.2 mRNA expression in the uninfected cells (*P* = 0.057).

## Discussion

Probiotics are one of the alternative strategies to feed antibiotics for prevention against infectious diseases. The beneficial roles of probiotics include production of antimicrobials, alteration of the intestinal microbiota, modulation of mucosal immunity and barrier function (Bermudez-Brito et al., 2012). *Lactobacillus* spp. are the most frequently used lactic acid bacteria as probiotic agents. They also showed species- and strain-specific characteristics in conferring probiotic benefits (Zheng et al., 2013; Valeriano et al., 2017). In the present study, we investigated the inhibitory effects of *L. acidophilus* and *L. fermentum* on the growth and α-toxin production of *C. perfringens*. Using primary intestinal epithelial cells of chicken embryos, we further examined the modulation of these lactobacilli on inflammatory signaling pathways in *C. perfringens*-infected cells.

It was demonstrated that probiotics outcompeted pathogens for nutrients and space by secreting inhibitory substances, such as organic acid, hydrogen peroxide and bacteriocins (Salminen et al., 2010; Messaoudi et al., 2013). We observed that *L. acidophilus* and *L. fermentum* greatly repressed the growth of *C. perfringens*, accompanied with the decrease of pH. Actually, *C. perfringens* was a pH sensitive bacterium. The lowest pH supporting the growth of *C. perfringens* type A isolates carrying chromosomal or plasmid-borne entertoxin genes was 5.1 ± 0.1 or 5.2 ± 0.1, respectively (Li and McClane, 2006). This was consistent with our finding that the terminal pH in the 20 h-fermentation of *C. perfringens* was 5.1.

To further demonstrate whether pH played an important role in inhibiting the growth of *C. perfringens*, we performed the agar well diffusion assay and broth culture inhibition assay. The pH-neutralized supernatants of lactobacilli cultures could not suppress the pathogen growth, which indicated that lower pH was the main factor. However, supernatants from lactobacilli cultures rather than MRS broth at pH 4.20 maintained inhibitory effects on the proliferation of *C. perfringens* until 48 h. This suggested that pH is not the only contributor and lactobacilli might secret antimicrobials, such as bacteriocins against *C. perfringens*. It was reported that both *L. acidophilus* and *L. fermentum* could produce antibacterial substances displaying a wide inhibitory spectrum including Gram-negative and Gram-positive pathogenic strains (Coconnier et al., 1997; Pascual et al., 2008).

The intestinal microflora forms a delicate ecosystem, in which the different bacterial species may regulate each other's growth and gene expression (Allaart et al., 2011). This regulation is achieved through the quorum sensing (QS) system, where inter- and intra-species communication occurs via released signal molecules (Bassler, 1999). It is clarified that the transcription of *plc*, the gene encoding the α-toxin, is modulated by the accessory gene regulator (*agr*) system (Ohtani et al., 2009; Ohtani and Shimizu, 2015). The autoinducing peptide encoded by *agr* is secreted into the environment and acts as a QS signal molecular resulting in α-toxin production. Recent studies demonstrated that *Lactobacillus* spp. or their secretions interfered the agr QS system mediated virulence gene expression of pathogens, such as *Staphylococcus aureus* (Li et al., 2011) and *C. difficile* (Yun et al., 2014). Therefore, we speculated that both *L. acidophilus* and *L. fermentum* reduced α-toxin production likely by quenching the similar system.

However, it could not be neglected that the environmental pH during lactobacilli fermentation also played a role on the toxin secretion of *C. perfringens*, which was evidenced by Allaart et al. (2011). Thus, we measured the α-toxin production in the absence of lactobacilli cells at different pHs (5.20 and 6.20). The lower environmental pH significantly decreased the production of α-toxin, suggesting that the acid secreted by lactobacilli had a clear inhibitory effect on *C. perfringens* α-toxin production during coculturing. Furthermore, lactobacilli exhibited the ability of degrading established α-toxin, which might be due to the acid production. Actually, early in 1964, Pivnick et al. reported that the production and stability of *C. perfringens* toxins were influenced by the pH of growth medium (Pivnick et al., 1964). The optimum pH for synthesis of α-toxin is 6.7, and α-toxin appears unstable, evidenced by the rapid synthesis and degradation. In the current study, the α-toxin levels varied greatly in different experiments, indicating the instability of α-toxin. Whether pH affected α-toxin production at the transcriptional or translational level needs further investigation.

The ability to bind to various components of the host extracellular matrix, including collagen, is a trait often observed in enteric pathogens (Wade et al., 2015). The adherent abilities of *C. perfringens* strains are crucial for the pathogenesis of avian NE. It was demonstrated that the ability of avian strains of *C. perfringens* binding to specific collagen types correlated with their virulence (Wade et al., 2015) and the putative adhesion-encoding gene *cnaA* involved in the adherence (Wade et al., 2016). To prevent the pathogenic infection, probiotic bacterial strains should be able to compete with pathogens to occupy their potential binding sites in the gut (Štyriak et al., 2003; Schillinger et al., 2005). Probiotics usually exhibit high affinity to mucus by specific adhesins and adhesion is one of the important criteria for the selection of probiotic strains (Ouwehand et al., 2001). It was demonstrated that certain strains of lactic acid bacteria significantly reduced the adhesion of *C. perfringens* to immobilized mucus isolated from canine jejunal chyme *in vitro* (Rinkinen et al., 2003).

In the present work, when *L. acidophilus* adhered to the intestinal epithelial cells in advance and with a dose 10 times that of *C. perfringens*, the adhesion of *C. perfringens* was inhibited by 30% and the cell cytotoxicity was not significantly affected. On the one hand, *L. acidophilus* might gain an advantage in the space competition for binding sites. The bacterial size of L. acidophilus (>1,000 nm) (Nagy et al., 2016) was greater than that of *L. fermentum* (500–1,000 nm) (Sintubin et al., 2009), which was could be directly observed using microscopy (data not shown). This might be the one reason why *L. fermentum* did not show the similar inhibitory effect on the adhesive ability of *C. perfringens*. On the other hand, the expression of *cnaA* in *C. perfringens* was also regulated by agr QS system (Ohtani and Shimizu, 2015), which might be obstructed by *L. acidophilus* or its metabolites. Overall, *L. acidophilus* decreased the adhesion of *C. perfringens* to chicken intestinal epithelial cells, which might weaken its virulence.

*Lactobacillus* spp. plays an important role in the regulation of intestinal homeostasis and immunity (van Baarlen et al., 2013; Valeriano et al., 2017). The anti-inflammatory effects of *Lactobacillus* spp. were largely reported in pathogen-infected broiler chickens (Cao et al., 2012; Wang et al., 2017). In the primary culture of chicken intestinal epithelial cells, we found that pre-incubation with *L. acidophilus* or *L. fermentum* decreased the *C. perfringens*-induced up-regulation of pro-inflammatory factors (IL-6, IL-8, iNOS, LITAF, IL-1β). The alleviation of inflammation by lactobacilli was accordance with their preventative effects on cell cytotoxicity caused by *C. perfringens* infection.

As a Gram-positive bacterium, the main cell wall component of *C. perfringens* is PGN. It could be recognized by TLR2.2 or NOD1 in chicken intestinal epithelial cells and thereby activates the NF-κB signaling pathway. Consequently, the inflammatory responses were induced (Lu et al., 2009; Guo et al., 2015). *Lactobacillus* spp. are Gram-positive bacteria as well, but a host cell response to a microorganism being probiotic or pathogenic depends on the combination of the distinct microorganism-associated molecular patterns (MAMPs) that can interact with the various pattern recognition receptors (PRRs) and associated co-receptors that fine-tune signaling, as well as the presence of other microbial effector molecules, such as toxins (Lebeer et al., 2010). Numerous studies demonstrated that *Lactobacillus* spp. reduced inflammation through the inactivation of NF-κB signaling pathway in intestinal epithelial cells challenged by infectious agents including pathogens (Yang et al., 2012; Borthakur et al., 2013; Chen et al., 2013). The present study suggested that *L. acidophilus* and *L. fermentum* not only decreased the transcription of NF-κB p65, but also suppressed the mRNA expression of TLR2.2 and NOD1, which could be induced by *C. perfringens* and play a vital role in the signaling transaction. Therefore, the reduction of pro-inflammatory factor expression was not unexpected.

## Conclusion

In *in vitro* co-culture system, both *L. acidophilus* and *L. fermentum* inhibited the growth and α-toxin production of *C. perfringens* mainly by lowering the environmental pH. In primary chicken intestinal cells, pre-treatment with both probiotics alleviated the *C. perfringens*-induced inflammation via down-regulation of TLR2.2 and NOD1 mRNA expression as well as inactivation of NF-κB p65. Furthermore, *L. acidophilus* suppressed the adhesion of *C. perfringens* to the intestinal epithelial cells. *L. acidophilus* and *L. fermentum* showed great potential to weaken the virulence of *C. perfringens* during the pathogeneses of NE in broiler chickens.

## Author contributions

SG, DL, and YG conceived and designed the experiments. SG, BZ, and ZL performed the experiments. SG and YL analyzed the experimental data. SG, YG, and BD wrote this paper. All authors read and approved the final manuscript.

### Conflict of interest statement

The authors declare that the research was conducted in the absence of any commercial or financial relationships that could be construed as a potential conflict of interest.
